# Impact of hyperbilirubinemia associated acute kidney injury on chronic kidney disease after aortic arch surgery: a retrospective study with follow-up of 1-year

**DOI:** 10.1186/s13019-022-01992-7

**Published:** 2022-09-29

**Authors:** Lin Lyu, Haicheng Song, Guodong Gao, He Dong, Pingping Liao, Ziying Shen, Hui Liu, Haichen Chu, Li Yuan

**Affiliations:** 1 Department of Anesthesiology, The Affiliated Hospital of Qingdao University, No. 16 Jiangsu Road, Qingdao, 266000 Shandong China; 2 Heart Center, Women and Children’s Hospital Affiliated to Qingdao University, Qingdao, 266034 Shandong China; 3 Department of Cardiopulmonary Bypass, State Key Laboratory of Cardiovascular Medicine, Fuwai Hospital, National Center for Cardiovascular Disease, Chinese Academy of Medical Science and Peking Union Medical College, Beijing, China; 4 Department of Geriatric Medicine, The Affiliated Hospital of Qingdao University, Qingdao, 266000 Shandong China; 5 Department of Anesthesiology, The Affiliated Hospital of Qingdao University, No. 59, Haier Road, Qingdao, 266100 Shandong China

**Keywords:** Hyperbilirubinemia, Acute kidney injury, Chronic kidney disease, Aortic arch surgery

## Abstract

**Background:**

Hyperbilirubinemia (HB) is a serious complication in aortic arch surgery, which is associated with acute kidney injury (AKI). The association between HB and chronic kidney disease (CKD) is unknown. The aim of this study was to investigate the impact of HB associated AKI on CKD after aortic arch surgery.

**Methods:**

We reviewed 284 patients who underwent aortic arch surgery from 2016 to 2020 in our hospital. AKI was defined as a 50% increase in sCr from baseline value within the first 7 postoperative days. HB was defined as total bilirubin > 51.3 μmol/L. Patients were divided into 3 groups based on AKI and HB: HB associated AKI (HB-AKI) group (AKI patients suffered HB within the first 7 postoperative days); AKI without HB group and Non-AKI group.

**Results:**

Follow-up for 204 patients ranged from 3 to 12 months. Kaplan–Meier analysis showed that the 1-year cumulative incidence of CKD was highest in HB-AKI (32.6%) than AKI without HB (17.8%) and Non-AKI (7.4%, log-rank test, p < 0.001), and the incidence of CKD was higher in HB group than that in Non-HB group (26.7% vs. 13.9%, log-rank test, p = 0.015). Preoperative sCr (HR 1.010, 95% CI 1.004–1.016, p = 0.001), AKI without HB (HR 2.887, 95% CI 1.133–7.354, p = 0.026) and HB-AKI (HR 4.490, 95% CI 1.59–12.933, p = 0.005) were associated with CKD during 1-year follow-up.

**Conclusions:**

Patients suffering HB associated AKI were at more increased odds of CKD than patients suffering AKI without HB after aortic arch surgery.

## Introduction

Acute kidney injury (AKI) is a very common (35–65%) and serious complication in aortic arch surgery, which is associated with the development of chronic kidney disease (CKD) [1, 2]. The unique characteristics of aortic arch surgery include cardiopulmonary bypass (CPB), deep hypothermic circulatory arrest (DHCA) or moderate hypothermic circulatory arrest (MHCA), high volumes of blood products and fluid transfusion, and high doses of vasoactive agents. These unique characteristics could alter the renal perfusion repeatedly and therefore induce cycles of ischemia and reperfusion and lead to AKI. The ischemia and reperfusion is considered to be the main reason for AKI and other factors may exacerbate AKI [3].

Hyperbilirubinemia (HB) remains a prevalent postoperative complication in aortic arch surgery [4]. Bilirubin is a metabolic end product of heme degradation by the liver. Hemolysis caused by CPB, hepatic hypoperfusion and perioperative blood transfusion may disrupt the bilirubin metabolism and lead to HB [4, 5]. HB is associated with AKI after CPB [5]. Our previous research found that high concentration of bilirubin decreased proximal tubular epithelial cell viability via pro-apoptotic in vitro, and HB aggravated renal ischemia reperfusion injury in rat model [6]. However, the association between HB and CKD in aortic arch surgery is unknown. We therefore investigated the impact of HB associated AKI on development of CKD after aortic arch surgery.

## Methods

This retrospective study was exempted from formal institutional review board of our hospital because these was no modified intervention or disclosure of personal information and the informed consent from patients was waived.

### Study population

Medical records of patients ≥ 18 years of age who underwent aortic arch surgery at Affiliated Hospital of Qingdao University between June 2016 and June 2020 were retrospectively reviewed (n = 306). The kidney transplant history (n = 3), unavailable preoperative serum creatinine (sCr, n = 6), unavailable 7-days postoperative sCr (n = 10), preoperative CKD history (n = 3) were excluded. Thus, 284 cases were included in this study.

### Data collection

Demographic information included age, sex, body mass index (BMI), smoking history, left ventricular ejection fraction (LVEF), previous medical history (hypertension, diabetes, cardiac operation and coronary heart disease), surgical procedure and basic laboratory characteristics. The intraoperative variables were extracted including CPB characteristics, surgery duration, received blood product and blood loss. Postoperative data included complications, tracheotomy, 30-day mortality, mechanical ventilation time and hospital stay.

### Definitions

Postoperative AKI was defined as a 50% increase in sCr from baseline value or initiation of continuous renal replacement therapy (CRRT) within the first 7 postoperative days [7]. Urine output was not available in our postoperative cohort, so the urine output criteria was not used in this study. AKI was staged by severity (stages 1–3) according to the KDIGO guideline [7]. The baseline sCr level was defined as the preoperative sCr closest to the start of surgery. HB was defined as total bilirubin level > 51.3 μmol/L (3 mg/dL) after surgery [8]. In order to investigate the effect of HB on postoperative AKI, the HB associated AKI (HB-AKI) group was defined as AKI patients suffered from HB within the first 7 postoperative days. AKI without HB group was AKI patients without HB within the first 7 postoperative days. Non-AKI group was patients without AKI within the first 7 postoperative days.

CKD was defined by at least two separate estimated glomerular filtration rate (eGFR) values ≤ 60 ml^−1^ min^−1^ 1.73 m^−2^ separated by an interval of at least 90 days from onset of AKI according to the KDIGO guidelines [9]. The eGFR was calculated by modification of diet in renal disease (MDRD) creatinine equation [10].

Previous heart surgery was defined as value or coronary artery bypass surgery before aortic arch surgery. Surgery was defined as urgent when performed on the day of diagnosis. The definition used for DHCA was cooling to 18–20 °C via arterial and venous pump lines, and the MHCA was cooling to 20–28 °C. Stroke was defined as the accident presence of neurologic deficits with confirmation of the diagnosis by neurologists and neuroimaging examination [11]. The systemic response to infection was termed sepsis [12]. Postoperative 30-day mortality was defined as death during initial hospitalization or within 30 days of surgery.

### Study end point

The primary end point of this study was the development of CKD after aortic arch surgery, and one-year cumulative incidence of CKD compared for the three groups.

### Operative procedures

Briefly, after invasive blood pressure monitor was performed in the left radial artery and left femoral artery, general anesthesia was induced routinely. The right axillary artery was the preferred inflow site for CPB. When the nasopharyngeal temperature reached 18–28 °C, the circulatory arrest and unilateral antegrade cerebral perfusion were instituted and the 3 vessels of the arch were cross-clamped. In the hemiarch replacement, the arch vessels were cross-clamped to prevent residual blood from interfering with the procedure. During the frozen elephant trunk (FET) procedure and total arch replacement using a 4-branched graft, distal reperfusion was initiated once the distal anastomosis was completed to minimize the duration of cerebral and spinal cord ischemia. After completion of the repair and adequate rewarming, the patient was weaned from CPB. The securing hemostasis and sternal closure were performed in a routine manner.

### Statistical analyses

Categorical variables were expressed as frequencies with percentages and compared with Chi-square test or Fisher’s exact test. Continuous variables were presented as mean ± standard deviation or median with interquartile range (IQR) according to statistical distribution. For comparison among the 3 groups (Non-AKI, AKI without HB and HB-AKI), a one-way ANOVA test was used for continuous variables. Variables with a p < 0.1 in univariate analysis or variables deemed important for clinical outcomes were further analyzed in multivariable Cox model. Cox proportional hazard models were used to identify variables related to CKD after aortic arch surgery. One-year cumulative incidence of CKD was compared among the 3 groups or between HB group and Non-HB group by Kaplan–Meier curves and statistical significance was assessed by the log-rank test.

All statistical analyses were performed using SPSS software (IBM Corp. Version 26. Armonk, NY). Kaplan–Meier curves of CKD among the 3 groups or between HB group and Non-HB group were portrayed by GraphPad Prism software (version 9.0; San Diego, CA, USA). Statistical significance was considered as a two-tailed p < 0.05.

## Results

The mean age of patients was 53.20 ± 11.08 years. 201(70.8%) of patients were male. The main surgical procedures were total arch replacement (75.4%), total arch replacement and FET (16.5%) and hemiarch replacement (8.1%). The urgent surgery accounted for 77.8%. The incidence of AKI and HB was 65.8% and 32.0% respectively, and HB-AKI accounted for 23.9%. The total 30–day mortality was 8.8%.

The preoperative total bilirubin level was highest in HB-AKI group than that in Non-AKI group and AKI without HB group (p < 0.001, Table 1). The CPB time (p = 0.017) and cross-clamp time (p = 0.041) were longest in HB-AKI group (Table 2). Blood loss was most in HB-AKI group (p = 0.004) and the HB-AKI group was received more blood transfusion during surgery. The incidence of 30-day mortality was highest in HB-AKI group (p = 0.024), and mechanical ventilation time was longest in HB- AKI group (p < 0.001, Table 3).

**Table 1:** Demographics of patients according to the study groups

Variable	Non-AKI (n = 97)	AKI without HB (n = 119)	HB-AKI (n = 68)	P
Age (y)	52.32 ± 11.36	52.77 ± 10.63	55.19 ± 11.35	0.225
BMI (kg/m^2^)	24.8 (22.9–27.0)	26.5 (29.1–24.1)	25.5 (23.7–28.4)	0.041
Male/Female	71/26	88/31	42/26	0.177
Hypertension (N, %)	55 (56.7%)	86 (72.3%)	45 (66.2%)	0.055
Diabetes (N, %)	6 (6.2%)	7 (5.9%)	9 (13.2%)	0.163
CHD (N, %)	10 (10.3%)	10 (8.4%)	6 (8.8%)	0.869
Smoking history (N, %)	46 (47.4%)	56 (47.1%)	23 (33.8%)	0.154
LVEF (%)	60.0 (60.0–61.0)	60.0 (60.0–61.0)	60.0 (60.0–62.0)	0.304
Previous heart surgery (N, %)	7 (7.2%)	8 (6.7%)	5 (7.4%)	1.000
Urgent surgery (N, %)	72 (74.2%)	97 (81.5%)	52 (76.5%)	0.449
Renal arteries arising from the false lumen (N, %)	43 (47.3%)	52 (43.7%)	20 (29.4%)	0.104
*Main surgical procedure*				
Total arch replacement (N, %)	72 (74.2%)	96 (80.7%)	46 (67.6%)	0.140
Total arch replacement and FET (N, %)	18 (18.6%)	15 (12.6%)	14 (20.6%)	0.295
Hemiarch replacement (N, %)	7 (7.2%)	8 (6.7%)	8 (11.8%)	0.487
*Concomitant procedures*				
Bentall (N, %)	58 (59.8%)	58 (48.7%)	43 (63.2%)	0.101
Ascending aorta replacement (N, %)	39 (40.2%)	62 (52.1%)	30 (44.1%)	0.204
CABG (N, %)	7 (7.2%)	13 (10.9%)	6 (8.8%)	0.677
Valve surgery (N, %)	8 (8.2%)	3 (2.5%)	4 (5.9%)	0.154
*Basic laboratory characteristics*				
AST (IU/L)	22.0 (17.3–37.0)	26.0 (19.0–51.9)	25.0 (16.0–40.0)	0.216
ALT (IU/L)	25.0 (16.0–36.0)	30.2 (20.0–49.3)	27.0 (19.3–39.0)	0.219
Total bilirubin (μmol/L)	21.8 (17.1–31.2)	18.6 (14.3–25.3)	24.2 (18.5–37.3)	< 0.001
Direct bilirubin (μmol/L)	7.7 (5.3–10.7)	5.8 (4.4–8.1)	7.9 (5.9–10.2)	0.151
Albumin (g/L)	37.28 ± 4.69	38.36 ± 4.32	36.63 ± 4.98	0.040
sCr (μmol/L)	80.7 (68.6–104.0)	77.4 (62.0–98.0)	74.8 (58.7–103.2)	0.477
BUN (μmol/L)	7.0 (5.4–8.5)	7.1 (5.4–8.7)	6.6 (5.0–9.3)	0.985

*AKI* acute kidney injury; *HB* hyperbilirubinemia; *BMI* body mass index; *CHD* coronary heart disease; *LVEF* left ventricular ejection fraction; *FET* frozen elephant trunk; *CABG* coronary artery bypass grafting; *AST* aspartate transaminase; *ALT* alanine aminotransferase; *sCr* serum creatinine; *BUN* blood urea nitrogen

**Table 2:** Intraoperative characteristics according to the study groups

Variable	Non-AKI (n = 97)	AKI without HB (n = 119)	HB-AKI (n = 68)	P
*CPB characteristics*				
CPB time, min	230.0 (200.5–279.5)	244.0 (216.0–289.0)	252.5 (227.5–298.8)	0.017
Crossclamp time, min	125.0 (107.5–154.5)	126.0 (103.0–154.0)	138.5 (119.0–168.5)	0.041
Circulatory arrest time, min	20.0 (16.0–24.5)	20.0 (15.0–25.0)	21.0 (17.0–27.0)	0.089
Nadir Nasopharyngeal temperature, °C	24.1 (22.6–25.0)	23.9 (22.9–25.0)	23.9 (22.4–24.8)	0.432
Nadir Rectal temperature, °C	25.36 ± 1.94	25.77 ± 1.60	25.24 ± 1.50	0.079
DHCA/MHCA	7/90	8/111	7/61	0.659
Surgery duration, h	8.0 (7.5–9.4)	8.5 (8.0–10.0)	8.5 (8.0–10.0)	0.165
Received red blood cell U	2.0 (0–4.0)	2.8 (0–4.0)	4.0 (1.8–6.3)	0.001
Received plasma, mL	750 (550–950)	790 (600–1140)	800 (585–1242.5)	0.130
Received cryoprecipitate, U	20.0 (15.0–20.0)	20.0 (16.0–20.0)	20.0 (20.0–20.0)	0.010
Received blood platelet, U	2.0 (1.0–2.0)	2.0 (1.0–2.0)	2.0 (1.4–2.0)	0.388
Received washed autologous blood, mL	800 (600–1100)	1000 (765–1350)	1150 (715–1700)	0.001
Blood loss, mL	1000 (800–1350)	1200 (1000–1600)	1450 (1000–2000)	0.004

*AKI* acute kidney injury; *HB* hyperbilirubinemia; *CPB* cardiopulmonary bypass; *DHCA* deep hypothermic circulatory arrest; *MHCA* moderate hypothermic circulatory arrest

**Table 3:** Postoperative outcomes according to the study groups

Variable	Non-AKI group (n = 97)	AKI without HB group (n = 119)	HB-AKI group (n = 68)	P
Sepsis	2 (2.1%)	6 (5.0%)	7 (10.3%)	0.071
Re-exploration for bleeding	5 (5.2%)	12 (10.1%)	4 (5.9%)	0.400
CRRT	0 (0)	58 (48.7%)	41 (60.3%)	< 0.001
Tracheotomy	7 (7.2%)	25 (21.0%)	16 (23.5%)	0.004
ECMO	0 (0)	2 (1.7%)	0 (0)	0.511
Stroke	1 (1.0%)	4 (3.4%)	4 (5.9%)	0.209
*AKI class*				< 0.001
AKI1	0 (0)	26 (21.8%)	6 (8.8%)	
AKI2	0 (0)	27 (22.7%)	10 (14.7%)	
AKI3	0 (0)	66 (55.5%)	52 (76.5%)	
*Postoperative laboratory characteristics*				
Peak AST, IU/L	60.0 (40.9–92.0)	92.0 (56.0–213.0)	189.5 (71.0–545)	0.011
Peak ALT, IU/L	42.0 (29.3–77.5)	67.0 (35.0–124.1)	119.9 (45.0–428.5)	0.022
Peak total bilirubin, μmol/L	38.7 (25.8–51.2)	31.9 (24.8–43.0)	69.9 (59.0–98.8)	< 0.001
Peak sCr, μmol/L	101.0 (86.6–130.0)	204.0 (135.3–358.9)	253.8 (176.8–329.7)	< 0.001
Peak BUN, μmol/L	13.2 (10.8–16.5)	19.2 (15.0–26.2)	20.8 (14.3–30.2)	< 0.001
30-day death	4 (4.1%)	10 (8.4%)	11 (16.2%)	0.024
Mechanical ventilation time, h	24.0 (20.0–60.0)	72.0 (24.0–144.0)	72.5 (48.0–187.5)	< 0.001
Hospital stay, d	23.0 (19.0–32.0)	25.5 (20.0–38.0)	28.0 (17.0–44.0)	0.049
CKD	6 (6.2%)	21 (17.6%)	19 (27.9%)	0.001

*AKI* acute kidney injury; *HB* hyperbilirubinemia; *CRRT* continuous renal replacement therapy; *ECMO* extracorporeal membrane oxygenation; *AKI1* 1.5–1.9 times baseline sCr; *AKI2* 2.0–1.9 times baseline sCr; *AKI3* 3 times baseline or initiation of CRRT; *AST* aspartate transaminase; *ALT* alanine aminotransferase; *sCr* serum creatinine; *BUN* blood urea nitrogen; *CKD* chronic kidney disease

**Fig. 1:**
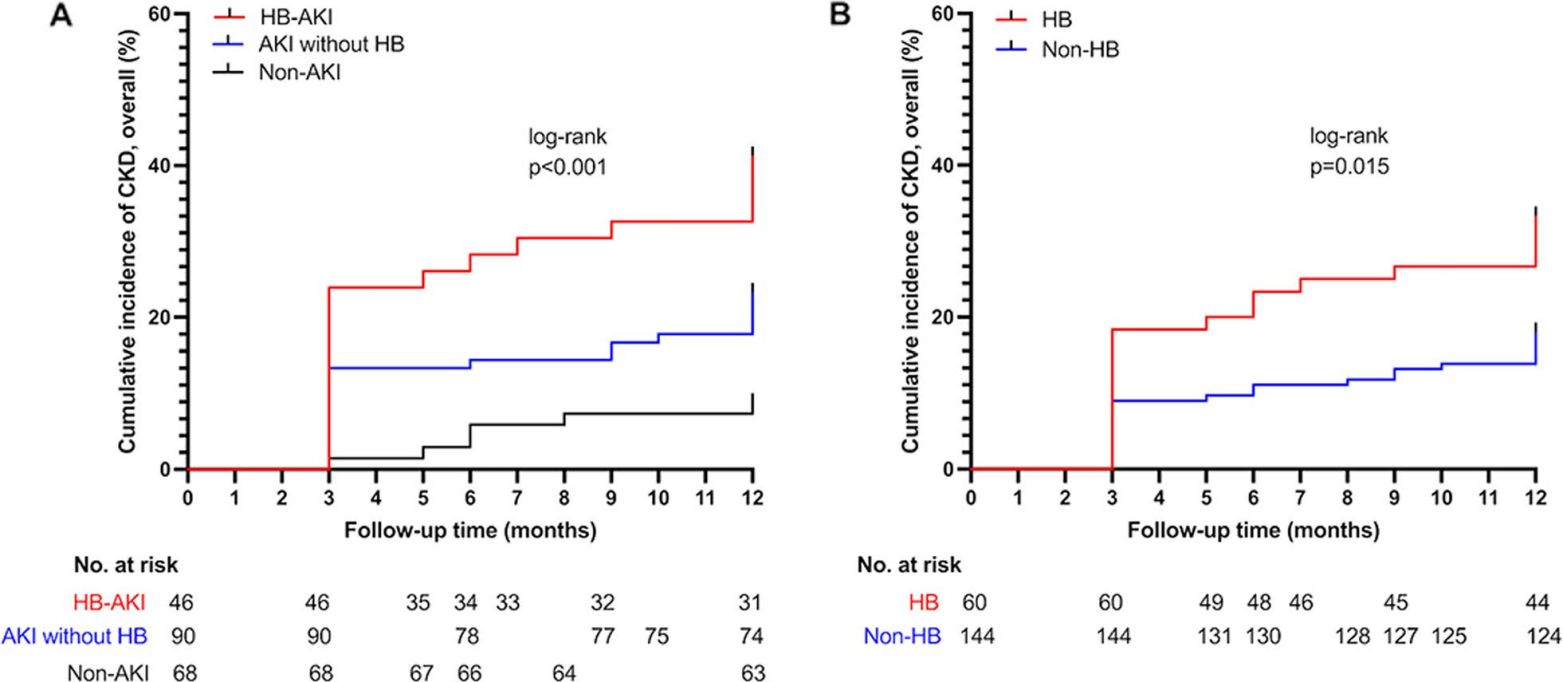
**A** Kaplan–Meier analysis curve of CKD after aortic arch surgery according to the study groups, **B** Kaplan–Meier analysis curve of CKD after aortic arch surgery compared between HB group and Non-HB group. AKI, acute kidney injury; HB, hyperbilirubinemia; CKD, chronic kidney disease

We exclude death cases (n = 29) in the first 3 months after surgery given that renal impairment must persist for 3 months to be defined as CKD, and the unavailable sCr during follow-up after surgery (n = 51) was also excluded. Follow-up for 204 patients ranged from 3 to 12 months. The mean follow-up duration was 10.66 ± 3.05 months. The cumulative incidence of CKD during 1-year follow-up period was 17.6%. Kaplan–Meier analysis showed that the 1-year cumulative incidence of CKD was highest in HB-AKI group (32.6%) than AKI without HB group (17.8%) and Non-AKI group (7.4%, log- rank test, p < 0.001, Fig. 1A). The 1-year cumulative incidence of CKD was higher in HB group than that in Non-HB group (26.7% vs. 13.9%, log-rank test, p = 0.015) by Kaplan–Meier analysis (Fig. 1B). After univariate analysis, Table 4 showed the results of Cox regression analysis for CKD during 1-year follow-up after surgery. Preoperative sCr (HR 1.010, 95% CI 1.004–1.016, p = 0.001), AKI without HB (HR 2.887, 95% CI 1.133–7.354, p = 0.026) and HB-AKI (HR 4.490, 95% CI 1.59–12.933, p = 0.005) were associated with CKD during 1-year follow-up after surgery.

**Table 4:** Univariable and multivariable cox regression analysis for postoperative CKD

	Univariable		Multivariable	
	HR (95% CI)	P	HR (95% CI)	P
Age (y)	1.024 (0.996–1.052)	0.088	1.020 (0.993–1.049)	0.153
BMI (kg/m^2^)	0.934 (0.860–1.014)	0.105		
Male/female	1.527 (0.839–2.779)	0.166		
Hypertension (N, %)	1.962 (0.974–3.954)	0.059	1.772 (0.856–3.666)	0.123
Diabetes (N, %)	1.885 (0.799–4.447)	0.148		
CHD (N, %)	1.778 (0.795–3.975)	1.161		
Smoking history (N, %)	1.075 (0.602–1.920)	0.808		
LVEF, %	0.983 (0.934–1.034)	0.499		
Previous heart surgery (N, %)	0.648 (0.157–2.672)	0.548		
Urgent surgery (N, %)	1.065 (0.528–2.145)	0.861		
Renal arteries arising from the false lumen (N, %)	1.033 (0.574–1.857)	0.915		
*Main surgical procedure*				
Total arch replacement (N, %)	0.934 (0.484–1.805)	0.840		
Total arch replacement and FET (N, %)	1.845 (0.729–4.669)	0.196		
Hemiarch replacement (N, %)	0.554 (0.248–1.239)	0.151		
*Concomitant procedures*				
Bentall (N, %)	1.108 (0.621–1.977)	0.727		
Ascending aorta replacement (N, %)	0.800 (0.448–1.426)	0.449		
CABG (N, %)	1.670 (0.405–6.889)	0.478		
Valve surgery (N, %)	0.573 (0.205–1.598)	0.287		
*Basic laboratory characteristics*				
AST (IU/L)	1.001 (1.000–1.001)	0.048	1.000 (0.999–1.000)	0.428
ALT (IU/L)	1.001 (1.000–1.001)	0.117		
Total bilirubin (μmol/L)	0.994 (0.970–1.018)	0.627		
Direct bilirubin (μmol/L)	0.963 (0.857–1.082)	0.522		
Albumin (g/L)	1.004 (0.945–1.067)	0.898		
sCr (μmol/L)	1.007 (1.003–1.011)	0.001	1.010 (1.004–1.016)	0.001
BUN (μmol/L)	1.054 (0.954–1.164)	0.299		
*CPB characteristics*				
CPB time (min)	1.000 (0.995–1.004)	0.934		
Crossclamp time (min)	0.996 (0.988–1.004)	0.350		
Circulatory arrest time (min)	0.997 (0.967–1.028)	0.847		
Nadir Nasopharyngeal temperature (°C)	0.967 (0.811–1.153)	0.708		
Nadir Rectal temperature (°C)	0.999 (0.836–1.193)	0.989		
DHCA/MHCA	1.270 (0.394–4.092)	0.689		
Surgery duration (h)	0.980 (0.825–1.164)	0.821		
Received red blood cell (U)	1.060 (0.981–1.145)	0.140		
Received plasma (ml)	1.000 (0.999–1.000)	0.810		
Received cryoprecipitate (U)	1.014 (0.977–1.053)	0.458		
Received blood platelet (U)	1.017 (0.757–1.366)	0.910		
Received washed autologous blood (mL)	1.000 (1.000–1.001)	0.426		
Blood loss (mL)	1.000 (1.000–1.001)	0.595		
Sepsis (N, %)	1.808 (0.561–5.829)	0.321		
Re-exploration for bleeding (N, %)	2.001 (0.848–4.721)	0.113		
*Postoperative laboratory characteristics*				
Peak total bilirubin (μmol/L)	1.009 (1.001–1.016)	0.018	1.006 (0.995–1.016)	0.299
Peak AST (IU/L)	1.000 (1.000–1.000)	0.167		
Peak ALT (IU/L)	1.001 (1.000–1.001)	0.007	1.001 (1.000–1.002)	0.265
Non-AKI (N, %)	–	0.001	–	0.018
AKI without HB (N, %)	2.798 (1.129–6.932)	0.026	2.887 (1.133–7.354)	0.026
HB-AKI (N, %)	5.406 (2.157–13.550)	< 0.001	4.490 (1.59–12.933)	0.005

*AKI* acute kidney injury; *HR* hazard ratio; *CI* confidence interval; *BMI* body mass index; *CHD* coronary heart disease; *LVEF* left ventricular ejection fraction; *FET* frozen elephant trunk; *CABG* coronary artery bypass grafting; *AST* aspartate transaminase; *ALT* alanine aminotransferase; *sCr* serum creatinine; *BUN* blood urea nitrogen; *CPB* cardiopulmonary bypass; *DHCA* deep hypothermic circulatory arrest; *MHCA* moderate hypothermic circulatory arrest; *HB* hyperbilirubinemia

## Discussion

In this retrospective study, we investigated the association between postoperative HB associated AKI and development of CKD after aortic arch surgery. The incidence of postoperative HB following aortic arch surgery was 32.0% in our study, which is higher than that in other cardiac surgery (8%-25%) [5, 13]. The longer duration of CPB, DHCA or MHCA and more blood product transfusion in aortic arch surgery cause more hemolysis and abnormal liver function, which may explain the higher incidence of postoperative HB [14, 15]. The hemolysis occurs and bilirubin is increasing gradually during CPB [16]. The postoperative peak total bilirubin level was at the second or third day after aortic arch surgery [4, 13], which was consistent with our study (data was not shown). Furthermore, although the majority of AKI occurs within the first 2–3 postoperative days according to the increase of sCr [17], diagnosis of the AKI delays using the elevation of sCr owing to the rate-limiting step of sCr production and release by skeletal muscle [18]. The renal has been injured since the CPB ended according to other biomarkers for predicting AKI [17]. Therefore, the impact of HB on renal during a window of time corresponding to the initiation or early extension phases of ischemia reperfusion injury in cardiac surgery with CPB was the same as HB aggravated renal ischemia reperfusion injury in rat model as our previous study reported [6]. The post-cardiac surgery AKI is associated with increased risk of CKD [2]. In order to investigate the effect of AKI exacerbated by HB on postoperative CKD, the HB-AKI group in our study was defined as AKI patients suffered from HB after surgery.

In our analyses, the CPB time and cross-clamp time were longer in HB-AKI group than that in Non-AKI group and AKI without HB group. The CPB time and cross-clamp time were risk factors of HB after cardiac surgery with CPB [5]. Received more blood transfusion and blood loss increase the bilirubin level, and they are risk factors of HB following cardiac surgery [5]. Moreover, CPB time, cross-clamp time, received more blood transfusion and blood loss were also associated with AKI after cardiac surgery with CPB [19, 20]. The similar risk factors of HB and AKI may explain the positive impact of HB on AKI and the poor outcomes of HB-AKI group in our study. The association between HB and AKI not only has been identified in cardiac surgery patients but also in other type of AKI patients. Wu et al. found that HB was an independent risk of contrast-related AKI following contrast-enhanced computed tomography [21].

Many studies have suggested that AKI are at considerable risk of developing CKD after cardiac surgery [2, 22]. We found that AKI patients had a 2.9-fold increased risk of CKD development than non-AKI patients during 1-year follow-up after surgery. However, HB-AKI patients had 4.5-fold higher risk of CKD compared to non-AKI patients, and the incidence of CKD was higher in HB-AKI group than that in AKI without HB group during 1-year follow-up. A cross-sectional analysis from National Health and Nutrition Examination Survey (NHANES) with a large adult cohort concluded that increasing serum concentration of total bilirubin was independent associated with decreasing eGFR [23]. We also found incidence of CKD was higher in patients with postoperative HB. Therefore, we speculated that HB aggravated AKI, and then the AKI aggravated by HB combined with HB per se made patients suffered more CKD after aortic arch surgery.

However, many studies report an inverse association between bilirubin and renal injury [24, 25]. For example, Sakoh et al. [25] found that lower serum bilirubin concentration was independently associated with adverse renal outcomes in patients with moderate to severe CKD, and high serum bilirubin level negatively associated with the incidence of end-stage renal disease in lgA nephropathy [24]. The contradictory effects of bilirubin on kidney can be explained by the different serum bilirubin concentrations. Mild elevation of serum bilirubin has renal protective function via inhibition of oxidative stress and apoptosis [26]. The mild elevation of bilirubin concentration of renal protection is often below 17.1 μmol/L in clinical studies [24, 25]. However, the cutoff levels of bilirubin for renal protection is controversial [27]. The serum bilirubin level in cardiac surgery is higher than that in above clinical studies, and the mean peak serum bilirubin concentration was 213.4 ± 149.4 μmol/L in aortic arch surgery [28]. High bilirubin level induces toxicity by apoptosis and oxidative stress. HB could contribute to tubular damage by stimulating the generation of oxygen free radicals from mitochondria [29].

## Study limitation

There are several limitations in our study. First, the retrospective single center study had the inherent potential for bias. The lost data at 1-year follow-up (20%) was more. Second, the eGFR was used to define the CKD. However, though CKD was defined by at least two separate eGFR, eGFR was based on a single measurement, which may fluctuate influenced by other factors such as medical therapy and living habit. We don’t have these data. The early CKD event may be missed on account of the lack of data on albuminuria, which is a strong independent predictor of kidney dysfunction [30]. Third, the mechanism of renal injury by HB in CPB patients remains to be explored.

## Conclusions

Patients suffering HB associated AKI were at more increased odds of CKD than patients suffering AKI without HB after aortic arch surgery. Further clinical and mechanistic studies are needed to confirm our findings and to better understand the harmful mechanism of HB associated AKI in CKD.

## Data Availability

The datasets used or analyzed during the current study are available from the corresponding author on reasonable request.

## Abbreviations

AKI: Acute kidney injury
CKD: Chronic kidney disease
HB: Hyperbilirubinemia
CPB: Cardiopulmonary bypass
DHCA: Deep hypothermic circulatory arrest
MHCA: Moderate hypothermic circulatory arrest
LVEF: Left ventricular ejection fraction
AST: Aspartate aminotransferase
ALT: Alanine aminotransferase
sCr: Serum creatinine
BUN: Blood urea nitrogen
eGFR: Estimated glomerular filtration rate
CRRT: Continuous renal replacement therapy
KDIGO: Kidney disease improving global outcomes
MDRD: Modification of diet in renal disease
FET: Frozen elephant trunk
BMI: Body mass index
CHD: Coronary heart disease
CABG: Coronary artery bypass grafting
